# Climate Endgame: Exploring catastrophic climate change scenarios

**DOI:** 10.1073/pnas.2108146119

**Published:** 2022-08-01

**Authors:** Luke Kemp, Chi Xu, Joanna Depledge, Kristie L. Ebi, Goodwin Gibbins, Timothy A. Kohler, Johan Rockström, Marten Scheffer, Hans Joachim Schellnhuber, Will Steffen, Timothy M. Lenton

**Affiliations:** ^a^Centre for the Study of Existential Risk, University of Cambridge, Cambridge CB2 1SB, United Kingdom;; ^b^Centre for the Study of Existential Risk, and Darwin College, University of Cambridge, Cambridge CB2 1SB, United Kingdom;; ^c^School of Life Sciences, Nanjing University, Nanjing 210023, China;; ^d^Cambridge Centre for Environment, Energy and Natural Resource Governance, University of Cambridge, Cambridge CB2 3QZ, United Kingdom;; ^e^Center for Health and the Global Environment, University of Washington, Seattle, WA 98195;; ^f^Future of Humanity Institute, University of Oxford, Oxford OX2 0DJ, United Kingdom;; ^g^Department of Anthropology, Washington State University, Pullman, WA 99164-4910;; ^h^Santa Fe Institute, Santa Fe, NM 87501;; ^i^Cluster of Excellence ROOTS – Social, Environmental, and Cultural Connectivity in Past Societies, Christian-Albrechts-Universität, Kiel, 24118 Germany;; ^j^Potsdam Institute for Climate Impact Research, 14473 Potsdam, Germany;; ^k^Department of Environmental Sciences, University of Wageningen, 6708PB Wageningen, The Netherlands;; ^l^Earth System Science Department, Tsinghua University, Beijing 100190, China;; ^m^Fenner School of Environment and Society, The Australian National University, Canberra, ACT 2601, Australia;; ^n^Global Systems Institute, University of Exeter, Exeter EX4 4QE, United Kingdom

**Keywords:** catastrophic climate change, climate change, Earth system trajectories, Anthropocene, tipping elements

## Abstract

Prudent risk management requires consideration of bad-to-worst-case scenarios. Yet, for climate change, such potential futures are poorly understood. Could anthropogenic climate change result in worldwide societal collapse or even eventual human extinction? At present, this is a dangerously underexplored topic. Yet there are ample reasons to suspect that climate change could result in a global catastrophe. Analyzing the mechanisms for these extreme consequences could help galvanize action, improve resilience, and inform policy, including emergency responses. We outline current knowledge about the likelihood of extreme climate change, discuss why understanding bad-to-worst cases is vital, articulate reasons for concern about catastrophic outcomes, define key terms, and put forward a research agenda. The proposed agenda covers four main questions: 1) What is the potential for climate change to drive mass extinction events? 2) What are the mechanisms that could result in human mass mortality and morbidity? 3) What are human societies' vulnerabilities to climate-triggered risk cascades, such as from conflict, political instability, and systemic financial risk? 4) How can these multiple strands of evidence—together with other global dangers—be usefully synthesized into an “integrated catastrophe assessment”? It is time for the scientific community to grapple with the challenge of better understanding catastrophic climate change.

How bad could climate change get? As early as 1988, the landmark Toronto Conference declaration described the ultimate consequences of climate change as potentially “second only to a global nuclear war.” Despite such proclamations decades ago, climate catastrophe is relatively under-studied and poorly understood.

The potential for catastrophic impacts depends on the magnitude and rate of climate change, the damage inflicted on Earth and human systems, and the vulnerability and response of those affected systems. The extremes of these areas, such as high temperature rise and cascading impacts, are underexamined. As noted by the Intergovernmental Panel on Climate Change (IPCC), there have been few quantitative estimates of global aggregate impacts from warming of 3 °C or above (1). Text mining of IPCC reports similarly found that coverage of temperature rises of 3 °C or higher is underrepresented relative to their likelihood (2). Text-mining analysis also suggests that over time the coverage of IPCC reports has shifted towards temperature rise of 2 °C and below https://agupubs.onlinelibrary.wiley.com/doi/full/10.1029/2022EF002876. Research has focused on the impacts of 1.5 °C and 2 °C, and studies of how climate impacts could cascade or trigger larger crises are sparse.

A thorough risk assessment would need to consider how risks spread, interact, amplify, and are aggravated by human responses (3), but even simpler “compound hazard” analyses of interacting climate hazards and drivers are underused. Yet this is how risk unfolds in the real world. For example, a cyclone destroys electrical infrastructure, leaving a population vulnerable to an ensuing deadly heat wave (4). Recently, we have seen compound hazards emerge between climate change and the COVID-19 pandemic (5). As the IPCC notes, climate risks are becoming more complex and difficult to manage, and are cascading across regions and sectors (6).

Why the focus on lower-end warming and simple risk analyses? One reason is the benchmark of the international targets: the Paris Agreement goal of limiting warming to well below 2 °C, with an aspiration of 1.5 °C. Another reason is the culture of climate science to “err on the side of least drama” (7), to not to be alarmists, which can be compounded by the consensus processes of the IPCC (8). Complex risk assessments, while more realistic, are also more difficult to do.

This caution is understandable, yet it is mismatched to the risks and potential damages posed by climate change. We know that temperature rise has “fat tails”: low-probability, high-impact extreme outcomes (9). Climate damages are likely to be nonlinear and result in an even larger tail (10). Too much is at stake to refrain from examining high-impact low-likelihood scenarios. The COVID-19 pandemic has underlined the need to consider and prepare for infrequent, high-impact global risks, and the systemic dangers they can spark. Prudent risk management demands that we thoroughly assess worst-case scenarios.

Our proposed “Climate Endgame” research agenda aims to direct exploration of the worst risks associated with anthropogenic climate change. To introduce it, we summarize existing evidence on the likelihood of extreme climate change, outline why exploring bad-to-worst cases is vital, suggest reasons for catastrophic concern, define key terms, and then explain the four key aspects of the research agenda.

## Worst-Case Climate Change

Despite 30 y of efforts and some progress under the United Nations Framework Convention on Climate Change (UNFCCC) anthropogenic greenhouse gas (GHG) emissions continue to increase. Even without considering worst-case climate responses, the current trajectory puts the world on track for a temperature rise between 2.1 °C and 3.9 °C by 2100 (11). If all 2030 nationally determined contributions are fully implemented, warming of 2.4 °C (1.9 °C to 3.0 °C) is expected by 2100. Meeting all long-term pledges and targets could reduce this to 2.1 °C (1.7 °C to 2.6 °C) (12). Even these optimistic assumptions lead to dangerous Earth system trajectories. Temperatures of more than 2 °C above preindustrial values have not been sustained on Earth’s surface since before the Pleistocene Epoch (or more than 2.6 million years ago) (13).

Even if anthropogenic GHG emissions start to decline soon, this does not rule out high future GHG concentrations or extreme climate change, particularly beyond 2100. There are feedbacks in the carbon cycle and potential tipping points that could generate high GHG concentrations (14) that are often missing from models. Examples include Arctic permafrost thawing that releases methane and CO_2_ (15), carbon loss due to intense droughts and fires in the Amazon (16), and the apparent slowing of dampening feedbacks such as natural carbon sink capacity (17, 18). These are likely to not be proportional to warming, as is sometimes assumed. Instead, abrupt and/or irreversible changes may be triggered at a temperature threshold. Such changes are evident in Earth’s geological record, and their impacts cascaded across the coupled climate–ecological–social system (19). Particularly worrying is a “tipping cascade” in which multiple tipping elements interact in such a way that tipping one threshold increases the likelihood of tipping another (20). Temperature rise is crucially dependent on the overall dynamics of the Earth system, not just the anthropogenic emissions trajectory.

The potential for tipping points and higher concentrations despite lower anthropogenic emissions is evident in existing models. Variability among the latest Coupled Model Intercomparison Project Phase 6 (CMIP6) climate models results in overlap in different scenarios. For example, the top (75th) quartile outcome of the “middle-of-the-road” scenario (Shared Socioeconomic Pathway 3-7.0, or SSP3-7.0) is substantially hotter than the bottom (25th) quartile of the highest emissions (SSP5-8.5) scenario. Regional temperature differences between models can exceed 5 °C to 6 °C, particularly in polar areas where various tipping points can occur (*SI Appendix*).

There are even more uncertain feedbacks, which, in a very worst case, might amplify to an irreversible transition into a “Hothouse Earth” state (21) (although there may be negative feedbacks that help buffer the Earth system). In particular, poorly understood cloud feedbacks might trigger sudden and irreversible global warming (22). Such effects remain underexplored and largely speculative “unknown unknowns” that are still being discovered. For instance, recent simulations suggest that stratocumulus cloud decks might abruptly be lost at CO_2_ concentrations that could be approached by the end of the century, causing an additional ∼8 °C global warming (23). Large uncertainties about dangerous surprises are reasons to prioritize rather than neglect them.

Recent findings on equilibrium climate sensitivity (ECS) (14, 24) underline that the magnitude of climate change is uncertain even if we knew future GHG concentrations. According to the IPCC, our best estimate for ECS is a 3 °C temperature rise per doubling of CO_2_, with a “likely” range of (66 to 100% likelihood) of 2.5 °C to 4 °C. While an ECS below 1.5 °C was essentially ruled out, there remains an 18% probability that ECS could be greater than 4.5 °C (14). The distribution of ECS is “heavy tailed,” with a higher probability of very high values of ECS than of very low values.

There is significant uncertainty over future anthropogenic GHG emissions as well. Representative Concentration Pathway 8.5 (RCP8.5, now SSP5-8.5), the highest emissions pathway used in IPCC scenarios, most closely matches cumulative emissions to date (25). This may not be the case going forward, because of falling prices of renewable energy and policy responses (26). Yet, there remain reasons for caution. For instance, there is significant uncertainty over key variables such as energy demand and economic growth. Plausibly higher economic growth rates could make RCP8.5 35% more likely (27).

## Why Explore Climate Catastrophe?

Why do we need to know about the plausible worst cases? First, risk management and robust decision-making under uncertainty requires knowledge of extremes. For example, the minimax criterion ranks policies by their worst outcomes (28). Such an approach is particularly appropriate for areas characterized by high uncertainties and tail risks. Emissions trajectories, future concentrations, future warming, and future impacts are all characterized by uncertainty. That is, we can’t objectively prescribe probabilities to different outcomes (29). Climate damages lie within the realm of “deep uncertainty”: We don’t know the probabilities attached to different outcomes, the exact chain of cause and effect that will lead to outcomes, or even the range, timing, or desirability of outcomes (, 30). Uncertainty, deep or not, should motivate precaution and vigilance, not complacency.

Catastrophic impacts, even if unlikely, have major implications for economic analysis, modeling, and society’s responses (31, 32). For example, extreme warming and the consequent damages can significantly increase the projected social cost of carbon (31). Understanding the vulnerability and responses of human societies can inform policy making and decision-making to prevent systemic crises. Indicators of key variables can provide early warning signals (33).

Knowing the worst cases can compel action, as the idea of “nuclear winter” in 1983 galvanized public concern and nuclear disarmament efforts. Exploring severe risks and higher-temperature scenarios could cement a recommitment to the 1.5 °C to 2 °C guardrail as the “least unattractive” option (34).

Understanding catastrophic climate scenarios can also inform policy interventions, including last-resort emergency measures like solar radiation management (SRM), the injection of aerosols into the stratosphere to reflect sunlight (35). Whether to resort to such measures depends on the risk profiles of both climate change and SRM scenarios. One recent analysis of the potential catastrophic risk of stratospheric aerosol injection (SAI) found that the direct and systemic impacts are under-studied (36). The largest danger appears to come from “termination shock”: abrupt and rapid warming if the SAI system is disrupted. Hence, SAI shifts the risk distribution: The median outcome may be better than the climate change it is offsetting, but the tail risk could be worse than warming (36).

There are other interventions that a better understanding of catastrophic climate change could facilitate. For example, at the international level, there is the potential for a “tail risk treaty”: an agreement or protocol that activates stronger commitments and mechanisms when early-warning indicators of potential abrupt change are triggered.

## The Potential for Climate Catastrophe

There are four key reasons to be concerned over the potential of a global climate catastrophe. First, there are warnings from history. Climate change (either regional or global) has played a role in the collapse or transformation of numerous previous societies (37) and in each of the five mass extinction events in Phanerozoic Earth history (38). The current carbon pulse is occurring at an unprecedented geological speed and, by the end of the century, may surpass thresholds that triggered previous mass extinctions (39, 40). The worst-case scenarios in the IPCC report project temperatures by the 22nd century that last prevailed in the Early Eocene, reversing 50 million years of cooler climates in the space of two centuries (41).

This is particularly alarming, as human societies are locally adapted to a specific climatic niche. The rise of large-scale, urbanized agrarian societies began with the shift to the stable climate of the Holocene ∼12,000 y ago (42). Since then, human population density peaked within a narrow climatic envelope with a mean annual average temperature of ∼13 °C. Even today, the most economically productive centers of human activity are concentrated in those areas (43). The cumulative impacts of warming may overwhelm societal adaptive capacity.

Second, climate change could directly trigger other catastrophic risks, such as international conflict, or exacerbate infectious disease spread, and spillover risk. These could be potent extreme threat multipliers.

Third, climate change could exacerbate vulnerabilities and cause multiple, indirect stresses (such as economic damage, loss of land, and water and food insecurity) that coalesce into system-wide synchronous failures. This is the path of systemic risk. Global crises tend to occur through such reinforcing “synchronous failures” that spread across countries and systems, as with the 2007–2008 global financial crisis (44). It is plausible that a sudden shift in climate could trigger systems failures that unravel societies across the globe.

**Fig. 1.**
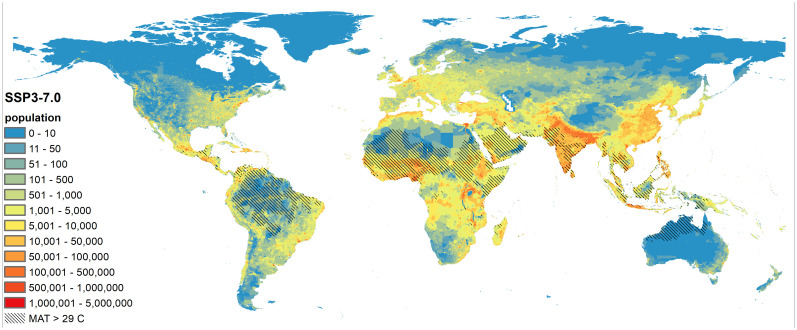
Overlap between future population distribution and extreme heat. CMIP6 model data [from nine GCM models available from the WorldClim database (45)] were used to calculate MAT under SSP3-7.0 during around 2070 (2060–2080) alongside Shared SSP3 demographic projections to ∼2070 (46). The shaded areas depict regions where MAT exceeds 29 °C, while the colored topography details the spread of population density.

The potential of systemic climate risk is marked: The most vulnerable states and communities will continue to be the hardest hit in a warming world, exacerbating inequities. Fig. 1 shows how projected population density intersects with extreme >29 °C mean annual temperature (MAT) (such temperatures are currently restricted to only 0.8% of Earth’s land surface area). Using the medium-high scenario of emissions and population growth (SSP3-7.0 emissions, and SSP3 population growth), by 2070, around 2 billion people are expected to live in these extremely hot areas. Currently, only 30 million people live in hot places, primarily in the Sahara Desert and Gulf Coast (43).

Extreme temperatures combined with high humidity can negatively affect outdoor worker productivity and yields of major cereal crops. These deadly heat conditions could significantly affect populated areas in South and southwest Asia(47).

**Fig. 2.**
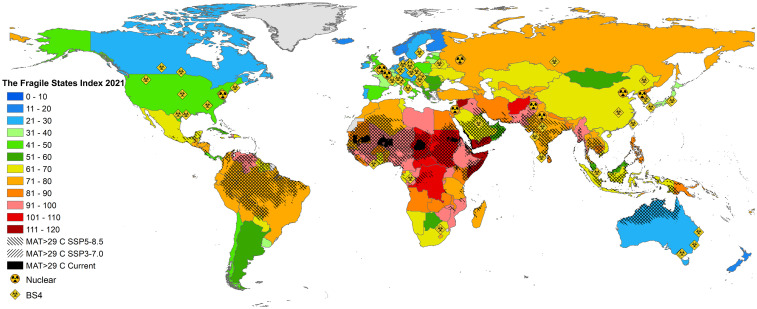
Fragile heat: the overlap between state fragility, extreme heat, and nuclear and biological catastrophic hazards. GCM model data [from the WorldClim database (45)] was used to calculate mean annual warming rates under SSP3-7.0 and SSP5-8.5. This results in a temperature rise of 2.8 °C in ∼2070 (48) for SSP3-7.0, and 3.2 °C for SSP5-8.5. The shaded areas depict regions where MAT exceeds 29 °C. These projections are overlapped with the 2021 Fragile State Index (FSI) (49). This is a necessarily rough proxy because FSI only estimates current fragility levels. While such measurements of fragility and stability are contested and have limitations, the FSI provides one of the more robust indices. This Figure also identifies the capitals of states with nuclear weapons, and the location of maximum containment Biosafety Level 4 (BS4) laboratories which handle the most dangerous pathogens in the world. These are provided as one rough proxy for nuclear and biological catastrophc hazards.

Fig. 2 takes a political lens on extreme heat, overlapping SSP3-7.0 or SSP5-8.5 projections of >29 °C MAT circa 2070, with the Fragile States Index (a measurement of the instability of states). There is a striking overlap between currently vulnerable states and future areas of extreme warming. If current political fragility does not improve significantly in the coming decades, then a belt of instability with potentially serious ramifications could occur.

Finally, climate change could irrevocably undermine humanity’s ability to recover from another cataclysm, such as nuclear war. That is, it could create significant latent risks (Table 1): Impacts that may be manageable during times of stability become dire when responding to and recovering from catastrophe. These different causes for catastrophic concern are interrelated and must be examined together.

**Table 1.** Defining key terms in the Climate Endgame agenda

Term	Definition
Latent risk	Risk that is dormant under one set of conditions but becomes active under another set of conditions.
Risk cascade	Chains of risk occurring when an adverse impact triggers a set of linked risks (3).
Systemic risk	The potential for individual disruptions or failures to cascade into a system-wide failure.
Extreme climate change	Mean global surface temperature rise of 3 °C or more above preindustrial levels by 2100.
Extinction risk	The probability of human extinction within a given timeframe.
Extinction threat	A plausible and significant contributor to total extinction risk.
Societal fragility	The potential for smaller damages to spiral into global catastrophic or extinction risk due to societal vulnerabilities, risk cascades, and maladaptive responses.
Societal collapse	Significant sociopolitical fragmentation and/or state failure along with the relatively rapid, enduring, and significant loss capital, and systems identity; this can lead to large-scale increases in mortality and morbidity.
Global catastrophic risk	The probability of a loss of 25% of the global population and the severe disruption of global critical systems (such as food) within a given timeframe (years or decades).
Global catastrophic threat	A plausible and significant contributor to global catastrophic risk; the potential for climate change to be a global catastrophic threat can be referred to as “catastrophic climate change”.
Global decimation risk	The probability of a loss of 10% (or more) of global population and the severe disruption of global critical systems (such as food) within a given timeframe (years or decades).
Global decimation threat	A plausible and significant contributor to global decimation risk.
Endgame territory	Levels of global warming and societal fragility that are judged sufficiently probable to constitute climate change as an extinction threat.
Worst-case warming	The highest empirically and theoretically plausible level of global warming.

## Defining the Key Terms

Although bad-to-worst case scenarios remain underexplored in the scientific literature, statements labeling climate change as catastrophic are not uncommon. UN Secretary-General António Guterres called climate change an “existential threat.” Academic studies have warned that warming above 5 °C is likely to be “beyond catastrophic” (50), and above 6 °C constitutes “an indisputable global catastrophe” (9).

Current discussions over climate catastrophe are undermined by unclear terminology. The term “catastrophic climate change” has not been conclusively defined. An existential risk is usually defined as a risk that cause an enduring and significant loss of long-term human potential (51, 52). This existing definition is deeply ambiguous and requires societal discussion and specification of long-term human values (52). While a democratic exploration of values is welcome, it is not required to understand pathways to human catastrophe or extinction (52). For now, the existing definition is not a solid foundation for a scientific inquiry.

We offer clarified working definitions of such terms in Table 1. This is an initial step toward creating a lexicon for global calamity. Some of the terms, such as what constitutes a “plausible” risk or a “significant contributor,” are necessarily ambiguous. Others, such as thresholding at 10% or 25% of global population, are partly arbitrary (10% is intended as a marker for a precedented loss, and 25% is intended as an unprecedented decrease; see *SI Appendix* for further discussion). Further research is needed to sharpen these definitions. The thresholds for global catastrophic and decimation risks are intended as general heuristics and not concrete numerical boundaries. Other factors such as morbidity, and cultural and economic loss, need to be considered.

We define risk as the probability that exposure to climate change impacts and responses will result in adverse consequences for human or ecological systems. For the Climate Endgame agenda, we are particularly interested in catastrophic consequences. Any risk is composed of four determinants: hazard, exposure, vulnerability, and response (3).

We have set global warming of 3 °C or more by the end of the century as a marker for extreme climate change. This threshold is chosen for four reasons: Such a temperature rise well exceeds internationally agreed targets, all the IPCC “reasons for concern” in climate impacts are either “high” or “very high” risk between 2 °C and 3 °C, there are substantially heightened risks of self-amplifying changes that would make it impossible to limit warming to 3 °C, and these levels relate to far greater uncertainty in impacts.

## Key Research Thus Far

The closest attempts to directly study or comprehensively address how climate change could lead to human extinction or global catastrophe have come through popular science books such as *The Uninhabitable Earth* (53) and *Our Final Warning* (10). The latter, a review of climate impacts at different degrees, concludes that a global temperature rise of 6 °C “imperils even the survival of humans as a species” (10).

We know that health risks worsen with rising temperatures (54). For example, there is already an increasing probability of multiple “breadbasket failures” (causing a food price shock) with higher temperatures (55). For the top four maize-producing regions (accounting for 87% of maize production), the likelihood of production losses greater than 10% jumps from 7% annually under a 2 °C temperature rise to 86% under 4 °C (56). The IPCC notes, in its Sixth Assessment Report, that 50 to 75% of the global population could be exposed to life-threatening climatic conditions by the end of the century due to extreme heat and humidity (6). *SI Appendix* provides further details on several key studies of extreme climate change.

The IPCC reports synthesize peer-reviewed literature regarding climate change, impacts and vulnerabilities, and mitigation. Despite identifying 15 tipping elements in biosphere, oceans, and cryosphere in the Working Group 1 contribution to the Sixth Assessment Report, many with irreversible thresholds, there were very few publications on catastrophic scenarios that could be assessed. The most notable coverage is the Working Group II “reasons for concern” syntheses that have been reported since 2001. These syntheses were designed to inform determination of what is “dangerous anthropogenic interference” with the climate system, that the UNFCCC aims to prevent. The five concerns are unique and threatened ecosystems, frequency and severity of extreme weather events, global distribution and balance of impacts, total economic and ecological impact, and irreversible, large-scale, abrupt transitions. Each IPCC assessment found greater risks occurring at lower increases in global mean temperatures. In the Sixth Assessment Report, all five concerns were listed as very high for temperatures of 1.2 °C to 4.5 °C. In contrast, only two were rated as very high at this temperature interval in the previous Assessment Report (6). All five concerns are now at “high” or “very high” for 2 °C to 3 °C of warming (57).

## A Sample Research Agenda: Extreme Earth System States, Mass Mortality, Societal Fragility, and Integrated Climate Catastrophe Assessments

We suggest a research agenda for catastrophic climate change that focuses on four key strands:

• Understanding extreme climate change dynamics and impacts in the long term
• Exploring climate-triggered pathways to mass morbidity and mortality
• Investigating social fragility: vulnerabilities, risk cascades, and risk responses
• Synthesizing the research findings into “integrated catastrophe assessments”

Our proposed agenda learns from and builds on integrated assessment models that are being adapted to better assess large-scale harms. A range of tipping points have been assessed (58–60), with effects varying from a 10% chance of doubling the social cost of carbon (61) up to an eightfold increase in the optimal carbon price (60). This echoes earlier findings that welfare estimates depend on fat tail risks (31). Model assumptions such as discount rates, exogenous growth rates, risk preferences, and damage functions also strongly influence outcomes.

There are large, important aspects missing from these models that are highlighted in the research agenda: longer-term impacts under extreme climate change, pathways toward mass morbidity and mortality, and the risk cascades and systemic risks that extreme climate impacts could trigger. Progress in these areas would allow for more realistic models and damage functions and help provide direct estimates of casualties (62), a necessary moral noneconomic measure of climate risk. We urge the research community to develop integrated conceptual and semiquantitative models of climate catastrophes.

Finally, we invite other scholars to revise and improve upon this proposed agenda.

### Extreme Earth System States.

We need to understand potential long-term states of the Earth system under extreme climate change. This means mapping different “Hothouse Earth” scenarios (21) or other extreme scenarios, such as alternative circulation regimes or large, irreversible changes in ice cover and sea level. This research will require consideration of long-term climate dynamics and their impacts on other planetary-level processes. Research suggests that previous mass extinction events occurred due to threshold effects in the carbon cycle that we could cross this century (40, 63). Key impacts in previous mass extinctions, such as ocean hypoxia and anoxia, could also escalate in the longer term (40, 64).

Studying potential tipping points and irreversible “committed” changes of ecological and climate systems is essential. For instance, modeling of the Antarctic ice sheet suggests there are several tipping points that exhibit hysteresis (65). Irreversible loss of the West Antarctic ice sheet was found to be triggered at ∼2 °C global warming, and the current ice sheet configuration cannot be regained even if temperatures return to present-day levels. At a 6 °C to 9 °C rise in global temperature, slow, irreversible loss of the East Antarctic ice sheet and over 40 m of sea level rise equivalent could be triggered (65). Similar studies of areas such as the Greenland ice sheet, permafrost, and terrestrial vegetation would be helpful. Identifying all the potential Earth system tipping elements is crucial. This should include a consideration of wider planetary boundaries, such as biodiversity, that will influence tipping points (66), feedbacks beyond the climate system, and how tipping elements could cascade together (67).

### Mass Morbidity and Mortality.

There are many potential contributors to climate-induced morbidity and mortality, but the “four horsemen” of the climate change end game are likely to be famine and undernutrition, extreme weather events, conflict, and vector-borne diseases. These will be worsened by additional risks and impacts such as mortality from air pollution and sea level rise.

These pathways require further study. Empirical estimates of even direct fatalities from heat stress thus far in the United States are systematically underestimated (68). A review of the health and climate change literature from 1985 to 2013 (with a proxy review up to 2017) found that, of 2,143 papers, only 189 (9%) included a dedicated discussion of more-extreme health impacts or systemic risk (relating to migration, famine, or conflict) (69). Models also rarely include adaptive responses. Thus, the overall mortality estimates are uncertain.

How can potential mass morbidity and mortality be better accounted for? 1) Track compound hazards through bottom-up modeling of systems and vulnerabilities (70) and rigorously stress test preparedness (71). 2) Apply models to higher-temperature scenarios and longer timelines. 3) Integrate risk cascades and systemic risks (see the following section) into health risk assessments, such as by incorporating morbidity and mortality resulting from a climate-triggered food price shock.

### Societal Fragility: Vulnerabilities, Risk Cascades, and Risk Responses.

More-complex risk assessments are generally more realistic. The determinants of risk are not just hazards, vulnerabilities, and exposures, but also responses (3, 72). A complete risk assessment needs to consider climate impacts, differential exposure, systemic vulnerabilities, responses of societies and actors, and the knock-on effects across borders and sectors (73), potentially resulting in systemic crises. In the worst case(s), a domino effect or spiral could continuously worsen the initial risk.

Societal risk cascades could involve conflict, disease, political change, and economic crises. Climate change has a complicated relationship with conflict, including, possibly, as a risk factor (74) especially in areas with preexisting ethnic conflict (75). Climate change could affect the spread and transmission of infectious diseases, as well as the expansion and severity of different zoonotic infections (76), creating conditions for novel outbreaks and infections (6,77). Epidemics can, in turn, trigger cascading impacts, as in the case of COVID-19. Exposure to ecological stress and natural disasters are key determinants for the cultural “tightness” (strictness of rules, adherence to tradition, and severity of punishment) of societies (78). The literature on the median economic damages of climate change is profuse, but there is far less on financial tail risks, such as the possibility of global financial crises.

Past studies could be drawn upon to investigate societal risk. Relatively small, regional climate changes are linked to the transformation and even collapse of previous societies (79, 80). This could be due to declining resilience and the passing of tipping points in these societies. There is some evidence for critical slowing down in societies prior to their collapse (81, 82). However, care is needed in drawing lessons from premodern case studies. Prehistory and history should be studied to determine not just how past societies were affected by specific climate hazards but how those effects differ as societies change with respect to, for example, population density, wealth inequality, and governance regime. Such framing will allow past and current societies to be brought under a single system of analysis (37).

**Fig. 3.**
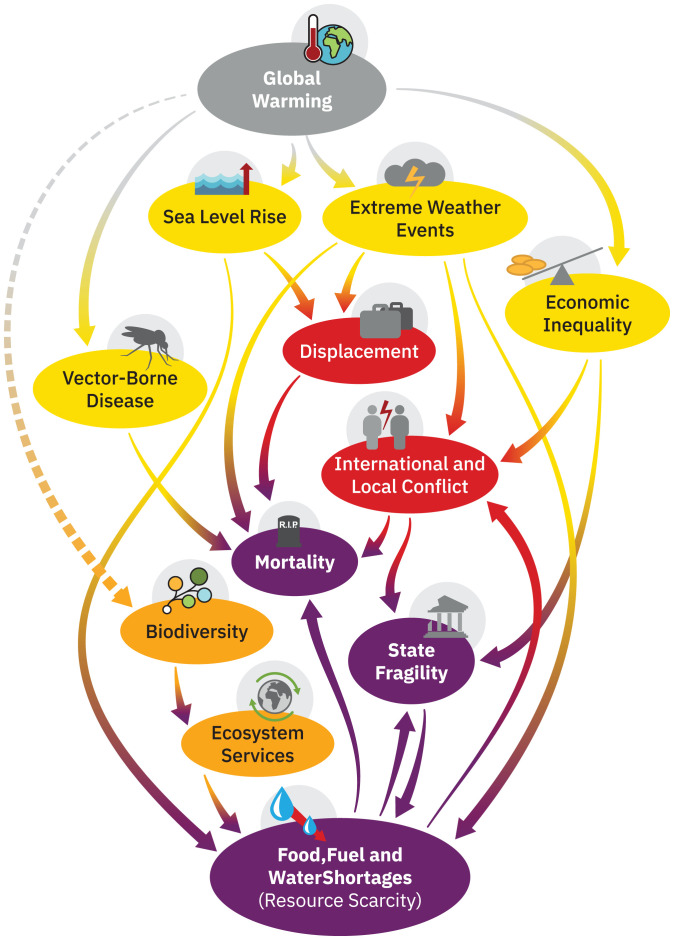
Cascading global climate failure. This is a causal loop diagram, in which a complete line represents a positive polarity (e.g., amplifying feedback; not necessarily positive in a normative sense) and a dotted line denotes a negative polarity (meaning a dampening feedback). See *SI Appendix* for further information.

The characteristics and vulnerabilities of a modern globalized world where food and transport distribution systems can buffer against traumas will need to feature in work on societal sensitivity. Such large, interconnected systems bring their own sources of fragility, particularly if networks are relatively homogeneous, with a few dominant nodes highly connected to everyone else (83). Other important modern-day vulnerabilities include the rapid spread of misinformation and disinformation. These epistemic risks are serious concerns for public health crises (84) and have already hindered climate action. A high-level and simplified depiction of how risk cascades could unfold is provided in Fig. 3.

### Integrated Catastrophic Assessments.

Climate change will unfold in a world of changing ecosystems, geopolitics, and technology. Could we even see “warm wars”—technologically enhanced great power conflicts over dwindling carbon budgets, climate impacts, or SRM experiments? Such developments and scenarios need to be considered to build a full picture of climate dangers. Climate change could reinforce other interacting threats, including rising inequality, demographic stresses, misinformation, new destructive weapons, and the overshoot of other planetary boundaries (85). There are also natural shocks, such as solar flares and high-impact volcanic eruptions, that present possible deadly synchronicities (86). Exploring these is vital, and a range of “standardized catastrophic scenarios” would facilitate assessment.

Expert elicitation, systems mapping, and participatory scenarios provide promising ways of understanding such cascades (73). There are also existing research agendas for some of these areas that could be funded (87).

Integration can be approached in several ways. Metareviews and syntheses of research results can provide useful data for mapping the interactions between risks. This could be done through causal mapping, expert elicitation, and agent-based or systems dynamics modeling approaches. One recent study mapped the evidence base for relationships between climate change, food insecurity, and contributors to societal collapse (mortality, conflict, and emigration) based on 41 studies (88).

A particularly promising avenue is to repurpose existing complex models to study cascading risks. The resulting network could be “stress tested” with standardized catastrophic scenarios. This could help estimate which areas may incur critical shortages or disruptions, or drastic responses (such as food export bans). Complex models have been developed to help understand past large-scale systemic disasters, such as the 2007–2008 global financial crisis (89). Some of these could be repurposed for exploring the potential nature of a future global climate crisis.

Systems failure is unlikely to be globally simultaneous; it is more likely to begin regionally and then cascade up. Although the goal is to investigate catastrophic climate risk globally, incorporating knowledge of regional losses is indispensable.

The potentially catastrophic risks of climate change are difficult to quantify, even within models. Any of the above-mentioned modeling approaches should provide a greater understanding of the pathways of systemic risk, and rough probabilistic guides. Yet the results could provide the foundation for argumentation-based tools to assess the potential for catastrophic outcomes under different levels of temperature rise (90). These should be fed into open deliberative democratic methods that provide a fair, inclusive, and effective approach to decision-making (91). Such approaches could draw on decision-making tools under uncertainty, such as the minimax principle or ranking decisions by the weighted sum of their best and worst outcomes, as suggested in the Dasgupta review of biodiversity (92).

## An IPCC Special Report on Catastrophic Climate Change

The IPCC has yet to give focused attention to catastrophic climate change. Fourteen special reports have been published. None covered extreme or catastrophic climate change. A special report on “tipping points” was proposed for the seventh IPCC assessment cycle, and we suggest this could be broadened to consider all key aspects of catastrophic climate change. This appears warranted, following the IPCC’s decision framework (93). Such a report could investigate how Earth system feedbacks could alter temperature trajectories, and whether these are irreversible.

A special report on catastrophic climate change could help trigger further research, just as the “Global warming of 1.5 °C” special report (94) did. That report also galvanized a groundswell of public concern about the severity of impacts at lower temperature ranges. The impact of a report on catastrophic climate change could be even more marked. It could help bring into focus how much is at stake in a worst-case scenario. Further research funding of catastrophic and worst-case climate change is critical.

Effective communication of research results will be key. While there is concern that fear-invoking messages may be unhelpful and induce paralysis (95), the evidence on hopeful vs. fearful messaging is mixed, even across metaanalyses (96, 97). The role of emotions is complex, and it is strategic to adjust messages for specific audiences (98). One recent review of the climate debate highlighted the importance of avoiding political bundling, selecting trusted messengers, and choosing effective frames (99). These kinds of considerations will be crucial in ensuring a useful and accurate civic discussion.

## Conclusions

There is ample evidence that climate change could become catastrophic. We could enter such “endgames” at even modest levels of warming. Understanding extreme risks is important for robust decision-making, from preparation to consideration of emergency responses. This requires exploring not just higher temperature scenarios but also the potential for climate change impacts to contribute to systemic risk and other cascades. We suggest that it is time to seriously scrutinize the best way to expand our research horizons to cover this field. The proposed “Climate Endgame” research agenda provides one way to navigate this under-studied area. Facing a future of accelerating climate change while blind to worst-case scenarios is naive risk management at best and fatally foolish at worst.

## Supplementary Material

Supplementary File

## Data Availability

Previously published data were used for this work (45, 46, 48, 49).
