# Supplementary material for: Climate Endgame: Exploring catastrophic climate change scenarios
Source: Proc Natl Acad Sci U S A. 2022 Aug 1;119(34):e2108146119. doi: 10.1073/pnas.2108146119 (PMC9407216; doi:10.1073/pnas.2108146119)
Supplement: Supplementary File [file pnas.2108146119.sapp.pdf]

Supporting Information

This file provides further details and figures for the overlap between population and extreme heat, the spread in model outcomes, elaboration on some of the provided definitions, further information on existing studies of extreme climate change and the potential for cascading climate failure.

Population and Extreme Heat

The overlap between population and extreme heat depicted in Figure 1 of the manuscript covers the emissions and population scenarios of SSP3-7.0. Here we present two related figures. Figure 1 provides a depiction of areas of extreme heat (a mean annual average temperature of 29 mean annual average temperature) for the emissions scenario SSP5-8.5 and population scenario of SSP5-8.

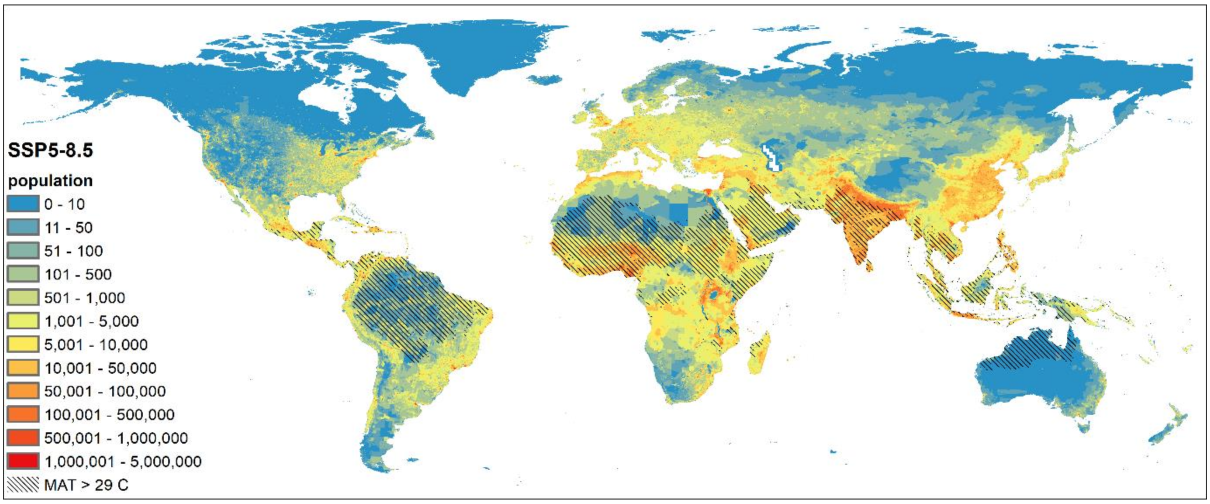

Figure 1: Overlap Between Future Population Distribution and Extreme Heat in SSP5-8.5

CMIP6 model data (from 9 GCM models available from the WorldClim database (1)*) were used to calculate MAT under SSP5-8.5 during around 2070 (2060-2080) alongside SSP5 demographic projections to approximately 2070* (2)*. The shaded areas depict regions where MAT exceeds 29°C, while the coloured topography details the spread of population density.*

The Box Plot in Figure 2 provides a comparison of the spread of model outcomes between these scenarios. SSP3-7.0 contains both a larger spread and a higher mean for populations under extreme heat, primarily due to comparatively higher population growth.

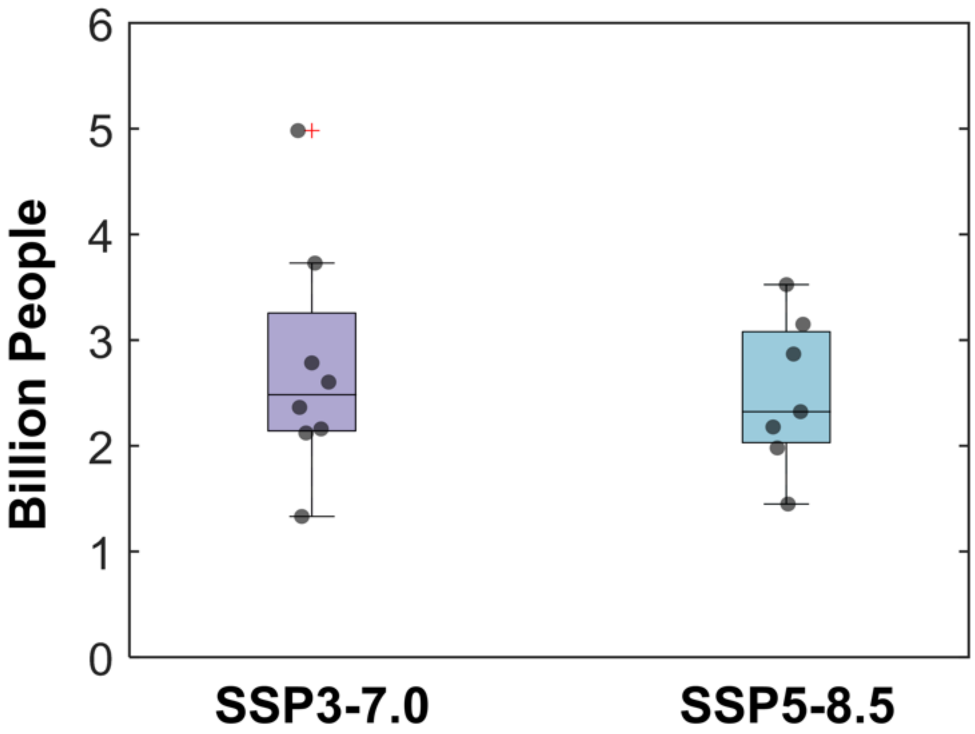

t

Figure 2: A Box Plot on the Overlap Between Population and Extreme Heat Scenarios

Model Spread

Figure 3 provides a comparison of warming by 2070 between the bottom and top quartile model outcomes. All listed temperatures denote mean annual average temperature. We used 20 datasets under the CMIP6 SSP5-8.5 (worst-case) scenario, all of which are publicly available through the Earth System Grid Federation data archive. The CMIP6 SSP5-8.5 are shown on the right-hand side. The left-hand diagrams depict model spread from 16 datasets under the CMIP6 SSP3-7.0 (middle of the road) scenario. The model spread can be greater than the difference between scenarios. For example, the top quartile outcome of SSP3-7.0 is substantially hotter than the bottom quartile of the worst-case SSP5-8.5 scenario. As shown in the third and final comparative diagram, regional variations in temperature between models can exceed 6°C, particularly in polar areas.

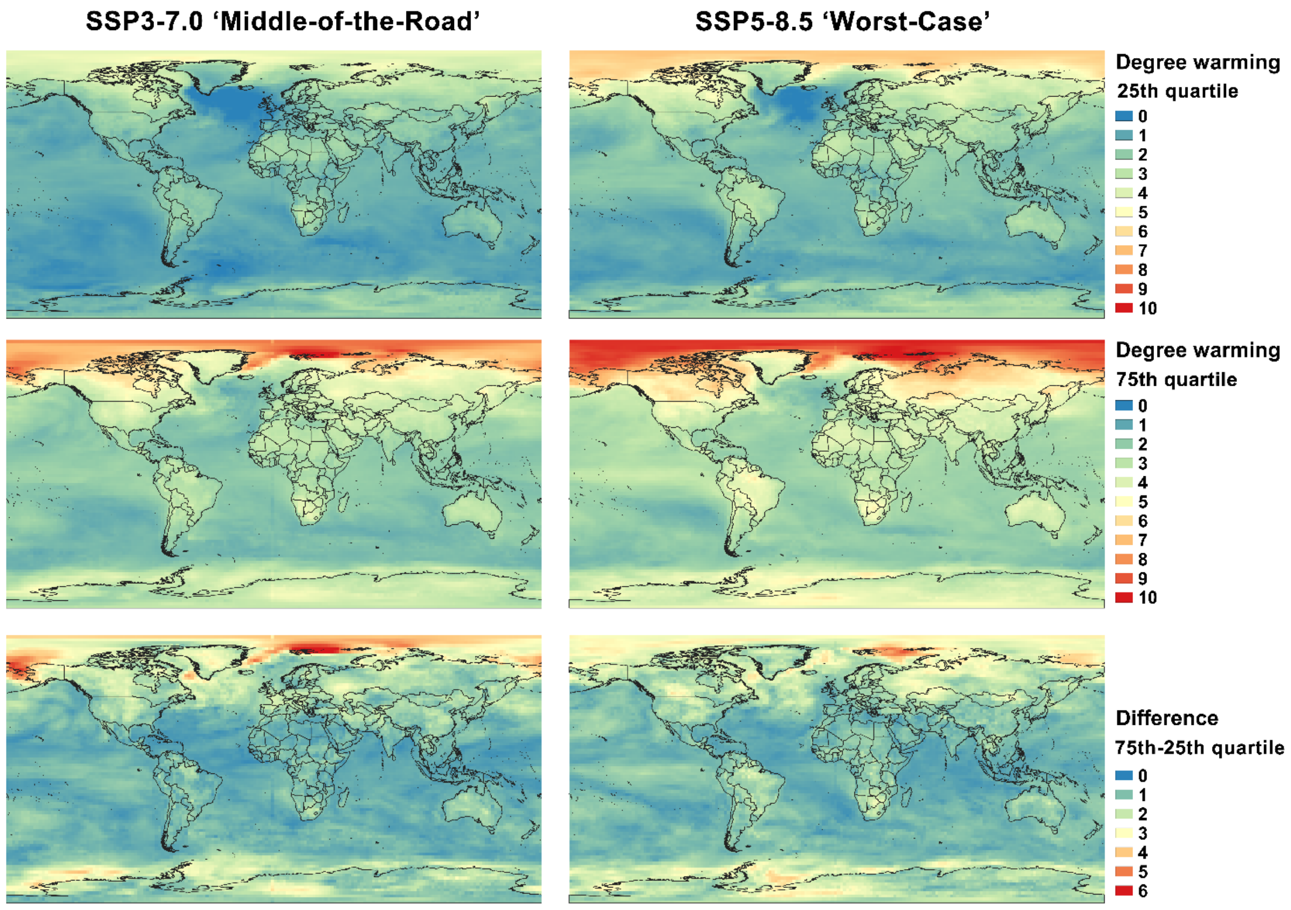

Figure 3. The Wide Spread in Model Outcomes

Definitions

Global Catastrophic Risk (GCR): *The probability of a loss of 25% of the global population and the severe disruption of global critical systems (such as food) within a given timeframe (years or decades). These should be seen as general heuristics rather than concrete thresholds. Other factors such as morbidity, economic, and cultural loss also need to be considered.*

The term 'Global Catastrophic Risk' (GCR) has never been conclusively defined. One of the most used definitions is a risk with the potential to create serious damage to global human well-being (3, 4). This has the benefit of ambiguity and the drawback of a lack of precision. Others have attempted to provide more specific quantitative thresholds, usually a loss of 10% of global population within a given timeframe (5). While this definition is more concrete, there is little justification for why 10% has been selected, and it neglects other undesirable outcomes. Avin et al link GCRs to the disruption of 'critical systems': systems or processes that "if disturbed beyond a certain limit or scale, could trigger a significant reduction in humanity's ability to survive in its current form (6)." Our definition blends together various aspects of these previous efforts. We adopt a 25% population loss threshold since this would be historically unprecedented with one or two high-uncertainty, potential exceptions such as the Toba volcanic eruption 74,000 years ago. We pair this with critical systems disruption to provide a more rounded definition.

Global Decimation Risk: The probability of a loss of 10% (or more) of global population and the severe disruption of global critical systems (such as food) within a given timeframe (years or decades). These should be seen as general heuristics rather than concrete thresholds. Other factors such as morbidity, economic, and cultural loss also need to be considered.

While 10% has often been used as the population loss threshold for a GCR, this is better described as a decimation risk. 'Decimation' in Latin means 'removal of a tenth'. It was used throughout history as a punishment in the military in which 1 in 10 soldiers in each cohort would be randomly executed. This acts as a useful proxy the worst global catastrophes with historical precedent. A few processes such as the colonisation of South and Central America (7), as well as the Black Death, and creation of the Mongol Empire all could have plausibly led to a 10% drop in global population, despite uncertainty in estimates. As with GCRs, we have combined this with a disruption in critical global systems to denote that more than a demographic bust is required to constitute a global catastrophe.

Societal Collapse: Significant socio-political fragmentation and/or state failure along with the relatively rapid, enduring, and significant loss capital, and systems identity. This can lead to large-scale increases in mortality and morbidity.

Definitions of societal collapse vary substantially. A popular and canonical definition is the rapid loss of a pre-established level of socio-political complexity (8). Others add a drastic loss of human population over an extended territory (9). One of the most recent formulations suggests a quick and enduring loss of systems identity as well as socio-ecological capital (10). These definitions suffer from several drawbacks. First, complexity is ambiguous, contested, and difficult to measure (10). Second, many historical collapses didn't involve large losses of population or ecological capital. Indeed, demographic busts, while tragic, often led to large-scale ecological restoration. In the case of the 'great dying' following the colonisation of the Americas, the loss of 56 million lives alongside the abandonment of multiple sites and breakdown of empires led to the reforestation of 55 million hectares and cooled the Earth by an average of 0.15°C (7). We centre the definition on socio-political fragmentation, since such disaggregation and state termination is the usual focus of collapse case studies (11) and the dramatic reduction of economic capital has been a consistent marker of collapse (12). Both are suitable for a future-orientated definition given that we live in a world dominated by nation-states and intense, global capital growth. Systems identity is a common feature in the socio-ecological literature and refers to the central features and structure that define a given system (10).

Sample Literature on Extreme Climate Change

In-depth investigations of the impacts of higher-temperature scenarios have begun. For example, a report from the Australian Academy of Science (AAS) highlights that 3°C of climate change in Australia carries wide-ranging impacts, such as rendering many businesses uninsurable and making ecosystems unrecognisable (13). The HELIX-EU (14) project focused on mapping the impacts of climate change at higher temperatures and the 2009 "*4 Degrees and Beyond International Climate Conference"* compiled papers relating to higher temperature scenarios (15). The World Bank's *Turn Down the Heat* series (16) concluded that likely impacts include severe reductions in crop yields, increased water scarcity, and unprecedented heat extremes (covering 70-80% of land surface area in North Africa, the Middle East, Latin America, and the Caribbean). Others have begun to map non-linear effects in a 4°C world (17). The common thread is that impacts increase, often non-linearly, with rising temperatures. For some systems, such as farming in sub-Saharan Africa, warming of 4°C, or less, could result in collapse or transformational change (15). While a useful start, such projects did not assess how impacts could contribute to global (or national) systemic risk. Data are lacking to answer the fundamental question of whether societies as we currently know them are compatible with extreme climate change (18). However, it appears likely that high enough temperatures are compatible with the current biosphere. One recent study of temperature thresholds for mass extinction events calculated that mass extinction events throughout the Phanerozoic have occurred by passing a temperature threshold of approximately >5.2 °C or a rate of >10 °C/Myr (19).

The closest coverage of catastrophic impacts under IPCC publications comes in the form of the Special Report on Managing the Risks of Extreme Events and Disasters to Advance Climate Change Adaptation (SREX).

Cascading Failure

Figure 3 of the manuscript is based on the perspectives of the authors. It is a high-level picture of how climate risk could unfold. There is a wealth of literature on many of these relationships and many have important caveats. The relationship between climate change and conflict is complex and depends on specific circumstances. However, a connection does appear to exist in cases where governance is weak (20, 21). Migration itself, and its impact on mortality and morbidity, is complex and effected by multiple factors beyond climatic change and sea level rise (22). How these relationships evolve under higher temperatures and greater resource and economic stress is unclear, but it seems intuitive that it would worsen, most likely in a non-linear fashion. Governance failures are one of the primary drivers in biodiversity loss (23). Global warming leads to increased economic inequality, particularly between countries (24). Inequality has a contested relationship with modern cases of civil war, and relevant studies have several limitations (25). However, there is evidence that wealth inequality has led to historical state failures and collapses through a variety of mechanisms (26, 27), a notion which intersects with both modern evidence of the socially corrosive effect of inequality (28, 29) and other theories of collapses, such as Structural Demographic Theory (30). These mechanisms include increased corruption, intra-elite competition, intra-societal conflict, and an impeded capacity to respond appropriately to impending crises (12). Shortages in food, fuel and water have been linked to numerous forms of conflict (31).

There is considerably more to the picture, including adaptive measures, the potential for nuclear war, and economic variables. For instance, sufficiently large injury and morbidity could potentially overwhelm health care services, as illustrated by the COVID-19 pandemic. Inclusion of these details should be coupled with closer geographical consideration as mortality rates and the costs of adaptation vary significantly across regions and countries (32).

References

1. WorldClim, WorldClim- Global Climate Data: Free Climate Data for Ecological Modeling and GIS (2019) (December 17, 2020).

2. International Institute for Applied Systems Analysis, SSP Database (Shared Socioeconomic

Pathways) - Version 2.0 (2019) (December 17, 2020).

3. N. Bostrom, M. M. Ćirković, Global catastrophic risks. 554 (2008).

4. S. J. Beard, *et al.*, Assessing climate change's contribution to global catastrophic risk. *Futures* **127** (2021).

5. GCF, "Global Catastrophic Risks Report 2018" (2018).

6. S. Avin, *et al.*, Classifying global catastrophic risks. *Futures* (2018) https:/doi.org/10.1016/j.futures.2018.02.001.

7. A. Koch, C. Brierley, M. M. Maslin, S. L. Lewis, Earth System Impacts of the European Arrival and Great Dying in the Americas After 1492. *Quat. Sci. Rev.* **207**, 13–36 (2019).

8. J. Tainter, *Collapse of Complex Societies*, 1st Ed. (Cambridge University Press, 1990).

9. J. Diamond, *Collapse: How Societies Choose to Fail or Succeed* (Penguin Books, 2011).

10. G. S. Cumming, G. D. Peterson, Unifying Research on Social–Ecological Resilience and Collapse. *Trends Ecol. Evol.* **32**, 695–713 (2017).

11. G. D. Middleton, *Understanding collapse: Ancient history and modern myths* (2017) https:/doi.org/10.1017/9781316584941.

12. L. Kemp, "Diminishing Returns on Extraction: How Inequality and Hierarchy Create Fragility" in *Historic Systemic Collapse*, M. A. Centeno, P. W. Callahan, P. Larcey, T. Patterson, Eds. (Routledge, 2022).

13. O. Hoegh-Guldberg, *et al.*, "The Risks to Australia of a 3oC World."

14. Tyndall Centre, High-End cLimate Impacts and eXtremes (HELIX) (2019) (December 6, 2019).

15. M. New, D. Liverman, H. Schroder, K. Anderson, Four Degrees and Beyond: The Potential for a Global Temperature Increase of Four Degrees and its Implications. *Philos. Trans. R. Soc. A Math. Phys. Eng. Sci.* **369**, 9–19 (2011).

16. World Bank, "Turn Down the Heat: Why a 4°C Warmer World Must Be Avoided" (2012).

17. H. Schellnhuber, *et al.*, "The Challenge of a 4°C World by 2100" in *Handbook on Sustainability Transition and Sustainable Peace*, (2016), pp. 267–283.

18. A. Jordan, *et al.*, Going Beyond Two Degrees? The Risks and Opportunities of Alternative Options. *Clim. Policy* **13**, 751–769 (2013).

19. H. Song, *et al.*, Thresholds of Temperature Change for Mass Extinctions. *Nat. Commun.* **12**, 4694 (2021).

20. G. J. Abel, M. Brottrager, J. Crespo Cuaresma, R. Muttarak, Climate, conflict and forced migration. *Glob. Environ. Chang.* **54**, 239–249 (2019).

21. IPCC, Part A: Global and Sectoral Aspects. (Contribution of Working Group II to the Fifth Assessment Report of the Intergovernmental Panel on Climate Change). *Clim. Chang. 2014 Impacts, Adapt. Vulnerability.* (2014).

22. I. Boas, *et al.*, Climate Migration Myths. *Nat. Clim. Chang.* **9**, 901–903 (2019).

23. T. Amano, *et al.*, Successful Conservation of Global Waterbird Populations Depends on Effective Governance. *Nature* **553**, 199–202 (2018).

24. N. S. Diffenbaugh, M. Burke, Global Warming has Increased Global Economic Inequality. *Proc. Natl. Acad. Sci. U. S. A.* **116**, 9808–9813 (2019).

25. M. Levitt, The Neglected Role of Inequality in Explanations of the Collapse of Ancient States. *Cliodynamics* **10**, 1–53 (2018).

26. L. Kemp, "Diminishing Returns on Extraction: How Inequality and Hierarchy Create Fragility" in

The Collapse of Complex Systems, M. A. Centeno, Ed. (Princeton University Press).

27. M. Levitt, *Why Did Ancient States Collapse?* (Access Archaeology, 2019).

28. R. Wilkinson, K. Pickett, *The Spirit Level: Why Greater Equality Makes Societies Stronger* (Bloomsbury Publishing, 2011).

29. R. Wilkinson, K. Pickett, *The Inner Level: How More Equal Societies Reduce Stress, Restore Sanity and Improve Everyone's Well-Being* (Penguin Books, 2020).

30. P. Turchin, S. A. Nefedov, *Secular Cycles* (Princeton University Press, 2009).

31. D. Natalini, G. Bravo, A. W. Jones, Global Food Security and Food Riots – An Agent-Based Modelling Approach. *Food Secur.* **11**, 1153–1173 (2017).

32. K. L. Ebi, *et al.*, Health Risks of Warming of 1.5 °c, 2 °c, and Higher, Above Pre-Industrial Temperatures. *Environ. Res. Lett.* **13**, 1–11 (2018).
